# Tactile Sensors for Robotic Applications

**DOI:** 10.3390/s20247009

**Published:** 2020-12-08

**Authors:** Salvatore Pirozzi

**Affiliations:** Dipartimento di Ingegneria, Università degli Studi della Campania “Luigi Vanvitelli”, Via Roma, 29, 81031 Aversa (CE), Italy; salvatore.pirozzi@unicampania.it; Tel.: +39-081-501-0433

In recent years, tactile sensing has become a key enabling technology to implement complex tasks by using robotic systems. For example, the successful execution of robotic grasping and manipulation tasks is strongly dependent on the knowledge of objects’ geometrical and physical characteristics, especially when objects are deformable and can change their shapes depending on their interaction with the environment. To this aim, robotic systems are more and more frequently equipped with sensorized grippers, which estimate the object’s features by using tactile sensors. Moreover, a safe and efficient physical Human Robot Interaction (pHRI) requires the knowledge of interaction forces and contact locations in order to perform cooperation and co-manipulation tasks and to limit damage from accidental impacts. This crucial information can be obtained through direct measurements by using an artificial sense of touch.

Very often, in grasping tasks, the object features can also be estimated by combining the tactile sensors with additional sensors, e.g., the vision systems and the six-axis force/torque sensors. Vision data are used more frequently due to the efficiency in data collection, but the vision alone may not be an efficient solution due to the difficulties in extracting the image features from a complex background. Many researchers have been working on integrating vision, force/torque and tactile data for object recognition for more than 30 years. Recent papers on this field concern object pose and shape estimation, combination of visual and tactile exploration procedures, estimation of surface features, match tactile features to visual maps, reconstruct contact force/torque from tactile data and object recognition by using cross-modal approaches.

It is evident that the number of different contexts in which the sense of touch, alone or in combination with other sensors, can be fundamental for the robotic systems of the future is high and growing. The aim of this Special Issue is to present robotic applications for which tactile sensing together with alternative sensing systems represent solutions that allow clear improvements for task automation.

## Summary of Special Issue

In [1], authors present the design and calibration of a new force/tactile sensor for dexterous manipulation. After the calibration, the device is able to estimate contact forces, torsional moment, object slip and object geometry: fundamental features when objects are fragile and deformable. Grasping experiments are reported to validate the proposed solution.

The combination of multi-sensor systems and control algorithms is another approach frequently used for robotic applications. The study in [2] proposes an intelligent real-time grasping system for the handling of objects with unknown properties in cluttered scenes, by combining different sensing system and control algorithms. Experiments with a five-finger robot hand are reported.

The estimation of slippage events, the sliding avoidance and stability of a grasp are fundamental aspects for advanced manipulation tasks. Many researchers, such as the authors of [3], continue to work on the development of technology solutions which allow improvements for these aspects. In particular, they proposed a solution based on a three-axis accelerometer embedded in a silicone chamber combined with a silicone rubber base. The design, the working principle and the fabrication procedure are detailed, and experiments are presented to demonstrate the effectiveness of the proposed approach in presence of sliding movements.

The authors of [4] propose the fusion of tactile data and vision data for estimating the pose of in-hand objects grasped with an underactuated robotic hand. These kind of data are very often combined with the aim to reproduce the human approach in manipulation. The data are used together with fuzzy logic controllers suitably designed to obtain stable grasps. Experiments are reported to demonstrate system capabilities.

New technologies are continuously investigated for the development of innovative tactile sensors. In [5], the authors present a novel type of soft resistive tactile sensor called “soft magnetic powdery sensor” (soft-MPS), which comprises ferromagnetic powder, immobilized in a liquid resin after orienting in a magnetic field. The proposed technology allows the realization of sensor units in any shape and to detect collisions in robot hands/arms or in ultra-sensitive touchscreen devices.

The authors of [6] exploit machine a learning approach to compensate hysteresis in a six-axis force sensor, in order to achieve high-precision measurements. The proposed approach allows to extension to multiple axis the hysteresis compensation typically applied only to one-axis sensors. Experimental results show the effectiveness of the proposed solution.

Many robotic tasks request the estimation of contact location with high precision. The paper [7] presents a design and characterization procedure for magnetic-based soft tactile sensors with the aim to locate a contact force application point. This procedure provides conditions under which it is possible to achieve the desired performance for the tactile sensor in identifying the contact location. An illustrative example demonstrates the efficacy of the proposed design procedure.

Development of human–machine interfaces, also for impaired people, is another research field that has been expanding recently. The paper [8] presents two methodologies for delivering multimedia content to visually impaired people with the use of a haptic device and braille display. The proposed approach uses 2D multiarray braille display to represent media content contour, illustrations and figures through quantization and binarization.

The exploitation of tactile data represents an open research field, very interesting in recent years. In paper [9], authors propose a novel method for active tactile perception based on 3D neural networks on the basis of tactile data available from a high-resolution tactile sensor installed on a robot gripper. An exploration procedure is performed in order to estimate information about both the external shape of the grasped object and internal features. A new 3D representation of tactile data is presented, by introducing an appropriate tensor used to feed a 3D Convolutional Neural Network, called 3D TactNet.

Manipulation by using anthropomorphic robotic and prosthetic hands still represents a research challenge. A fundamental aspect concerns the integration of a sensory system for control purposes in the limited space available. The authors of [10] present a scalable design model of artificial fingers, which combines mechanical design and a multi-modal sensor system for the measurement of normal and shear force, distance, acceleration, temperature, and joint angles. The design is fully parametric, allowing automated scaling of the fingers to arbitrary dimensions in the human hand spectrum. Different physical demonstrators are presented to demonstrate the effectiveness of the proposed approach.

Taking a cue from the animal world alternative solutions to the touch are represented by vibrissae. Animals like mice and rats use vibrissae in order to detect different features, e.g., object-distances and object-shapes. In [11], the authors deal with the effect of multi-point contacts in a specific scanning scenario, where an artificial vibrissa is swept along an object contours. They propose a model to simulate vibrissae during an object scanning and also experiments to validate the simulation results.

The author of [12] presents a dual-function wearable device (Tacsac) with capacitive tactile sensing and integrated tactile feedback capability to enable communication among deafblind people. Tacsac comprises two main modules: the touch-sensing module and the vibrotactile module. The Tacsac device has been tested for independent sensing and actuation as well as a dual sensing-actuation mode. A mobile application was also developed to demonstrate the application of Tacsac for communication between deafblind person wearing the device and a mobile phone user who is not deafblind.
